# Antioxidant and Neuroprotective Effect of a Grape Pomace Extract on Oxaliplatin-Induced Peripheral Neuropathy in Rats: Biochemical, Behavioral and Histopathological Evaluation

**DOI:** 10.3390/antiox11061062

**Published:** 2022-05-27

**Authors:** Chryssa Bekiari, Fotios Tekos, Zoi Skaperda, Aikaterini Argyropoulou, Alexios-Leandros Skaltsounis, Demetrios Kouretas, Anastasia Tsingotjidou

**Affiliations:** 1 Laboratory of Anatomy, Histology and Embryology, School of Veterinary Medicine, Faculty of Health Sciences, Aristotle University of Thessaloniki, 54124 Thessaloniki, Greece; chmpekia@vet.auth.gr; 2 Laboratory of Animal Physiology, Department of Biochemistry and Biotechnology, University of Thessaly, 41500 Larissa, Greece; ftekos@uth.gr (F.T.); zoskaper@bio.uth.gr (Z.S.); dkouret@uth.gr (D.K.); 3 Division of Pharmacognosy and Natural Products Chemistry, Department of Pharmacy, National Kapodistrian University of Athens, Panepistimioupoli, Zografou, 15771 Athens, Greece; katarg@pharm.uoa.gr (A.A.); skaltsounis@pharm.uoa.gr (A.-L.S.)

**Keywords:** oxaliplatin, peripheral neuropathy, rat, grape pomace, DRG, behavioral tests, parvalbumin, GFAP

## Abstract

Oxaliplatin is a widely used chemotherapeutic agent. Despite its many beneficial aspects in fighting many malignancies, it shares an aversive effect of neuropathy. Many substances have been used to limit this oxaliplatin-driven neuropathy in patients. This study evaluates the neuroprotective role of a grape pomace extract (GPE) into an oxaliplatin induced neuropathy in rats. For this reason, following the delivery of the substance into the animals prior to or simultaneously with oxaliplatin, their performance was evaluated by behavioral tests. Blood tests were also performed for the antioxidant activity of the extract, along with a histological and pathological evaluation of dorsal root ganglion (DRG) cells as the major components of the neuropathy. All behavioral tests were corrected following the use of the grape pomace. Oxidative stressors were also limited with the use of the extract. Additionally, the morphometrical analysis of the DRG cells and their immunohistochemical phenotype revealed the fidelity of the animal model and the changes into the parvalbumin and GFAP concentration indicative of the neuroprotective role of the pomace. In conclusion, the grape pomace extract with its antioxidant properties alleviates the harmful effects of the oxaliplatin induced chronic neuropathy in rats.

## 1. Introduction

Oxaliplatin [(trans–1) 1,2–diaminocyclohexaneoxalatoplatinum (II)] is a highly active, third-generation platinum chemotherapeutic agent, widely used against advanced colorectal cancer and as an alternative option to cisplatin in ovarian [1,2], pancreatic [3,4], breast [5,6], lung [7] and lately in prostate [8] cancer. Oxaliplatin, like all platines, induces mild hematological (myelosuppression) and gastrointestinal side effects but it has advantages among other widely used platinum-based drugs, such as cisplatin and carboplatin, for its greater anti-tumor activity, absence of nephrotoxicity and reduced ototoxicity [9,10].

Oxaliplatin-based chemotherapy leads to severe neurotoxicity characterized clinically by two different types of symptoms [11]. The first type, is an acute neurosensory toxicity present in 90% of patients immediately or shortly after infusion, mainly characterized by dysesthesia and/or paresthesia of the distal extremities enhanced by exposure to cold [12]. Although this type of neuropathy is always reversible, long-term administration of oxaliplatin is associated with a chronic cumulative peripheral neuropathy (PN), seen as superficial and deep sensory loss, sensory ataxia, functional impairment, proprioceptive loss and decreased tendon reflexes [12,13,14]. It may last for several months after the end of oxaliplatin treatment and usually symptoms that emerge are so intense it results in discontinuation of the treatment [14,15,16].

Up until now, several researchers have reported that severe damage in Dorsal Root Ganglion (DRG) neurons leads to the development of PN [17,18,19,20]. In particular, it was found that oxaliplatin causes the selective atrophy of a subpopulation of dorsal root ganglion neurons, large DRG neurons (≥1000 μm^2^), without interfering with total DRG neuronal cell number [21].

Although DRG neurons are widely accepted to be the primary drug-target, several hypotheses have been proposed as possible pathogenetic mechanisms leading to PN. These include high levels of platinum-DNA adducts in DRG neurons compared to other cell tissues [22] prolonged activation of voltage-gated Na^+^ driving to excess Ca2^+^ influx [23] or sodium channel subtype Na_V_1.6 resurgent [24] and neuron energy failure due to mitochondrial DNA-platinum binding [25]. However, in recent studies, oxidative stress markers at lipid, protein and DNA levels were evident in plasma and tissues of the nervous system of rats after oxaliplatin-treatment [26], suggesting that oxidative stress can be an exceptionally important pathological mechanism [27] leading to all these observed alterations.

Use of antioxidant compounds, such as vitamin E [28,29], glutathione [30] and N-acetylcysteine [31,32] and, recently, antioxidant molecules such as the novel BPF-15 [33] and a-lipoic acid [32] along with oxaliplatin treatment has been found to have beneficial effects in induced PN. The use of natural occurring antioxidant agents, such as polyphenol and catechins rich green tea [34], polyphenol rich curcumin, isolated from the turmeric plant *Curcuma longa* [35,36] and cilibinin, found in seeds of the milk thistle (*Silybum marianum*) [26] were also proven to have significant protective effects towards evoked PN.

In this study, we evaluated the efficacy of a polyphenol-rich grape extract obtained from pomace for prevention of oxaliplatin associated PN in rats. The extract’s compounds, such as catechin and epicatechin and gallic acid have been proven to have potent neuroprotective [37], chemopreventive [38] and antioxidant [39] properties. Antioxidant effect of grape pomace extract (GPE) has also been verified to spinal cord neurotoxicity [40] and to bovine spermatozoa oxidative damage [41]. Recently, a polyphenol-rich grape pomace extract was found to exert a neuroprotective effect on hypothalamic neurons that were challenged with an oxidative stimulus [42]. The possible neuroprotective effect of the grape extract against platinum-induced histopathological damage in DRG neurons was tested. Furthermore, behavioral evaluation of the animals to reveal the potential clinically observed benefits was conducted.

## 2. Materials and Methods

### 2.1. Animals and Experimental Design

A total of 30 adult four-month-old female Wistar rats from our own colony, weighing between 160 and 260 gr at the start of experimentation were used in this study. During the experiment, animals were housed in spacious open cages with three animals per cage at the animal research facility of the Laboratory of Anatomy, Histology and Embryology, School of Veterinary Medicine, Faculty of Health Sciences, Aristotle University of Thessaloniki (EL-54-BIOexp-23). Experimentation received the approval of the Veterinary Directorate of Thessaloniki, and all experimental procedures and protocols were in accordance with the European Directive 86/609/EEC on the protection of animals used for scientific purposes since the new one, the Directive 2010/63/EU on the protection of animals used for scientific purposes EU, was adopted on 22 September 2010 and the experiments took place in 2008–2009. All animals had *ad libitum* access to standard rodent food 4RF25 from Mucedola (Scobis, Settimo Milanese, Milan, Italy) and water. The general condition of the animals was evaluated daily, and body weight was recorded twice a week, before each oxaliplatin administration. Animals’ health was monitored according to FELASA recommendations using IDEXX GmbH (IDEXX BioAnalytics, Kornwestheim, Germany) testing. Access to the facility was controlled. Room temperature of 20–22 °C, and humidity of 45–55%, were checked daily. A stable temperature was ensured by a HVAC system controlled by a thermostat. Lights were less than 325 lux and were programmed for 12 h:12 h light to dark. Noise was kept <85 db and there was no ultrasonic noise or vibration. The sanitation was undertaken every week by experienced personnel. An ear identification method was used in all animals.

**Figure 1:**
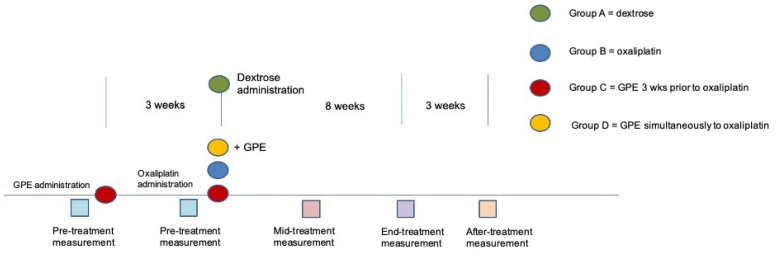
Diagram depicting the experimental protocol and the time points of the measurements.

Animals were assigned, by random selection, to one of four groups, A, B, C or D. Group A animals (*n* = 5) were used as dextrose control group (125 μL dextrose 5% i.p. twice a week) while group B (*n* = 5), C and D animals received oxaliplatin treatment at a dose of 3 mg/kg i.p. biweekly for eight weeks. Groups C and D were provided with GPE in water at a level of 75 mg per day per animal for eleven weeks (group C; *n* = 10; GPE pre-treatment was administered for an additional 3-week period before oxaliplatin treatment) or eight weeks (group D; *n* = 10; GPE and oxaliplatin treatment started simultaneously) (Figure 1).

### 2.2. Oxaliplatin Preparation

Oxaliplatin (Eloxatine, Sanofi Winthrop, Le Trait, France) was made up for injection in 5% dextrose to obtain a solution of 5 mg/mL and was aliquoted in eppendorfs of 125 μL, to obtain a stock solution. Eppendorfs were kept frozen at −12 °C until use.

### 2.3. Grape Pomace Extract Preparation

Grape pomace was supplied by Union of Santorini Cooperatives, SantoWines. The raw material was dried in a shady, well-ventilated place and extracted with water 100% at 45 °C for 4 h. In continuation, the water extract was treated with absorption resin chromatography (Amberlite XAD4, Supelco, Bellefonte, PA, USA) to remove sugars and to obtain a fraction rich in phenolic compounds. The polyphenolic rich fraction was recovered using analytical grade isopropanol (Fischer Scientific, Pittsburg, PA, USA). The fraction was dried in a rotary evaporator and lyophilized for complete solvent removal.

### 2.4. UHPLC-HRMS Analysis of the Grape Extract

UPLC-HRMS analysis was performed on an AQUITY system (Waters) connected to an LTQ-OrbitrapR XL hybrid mass spectrometer (Thermo Scientific, Waltham, MA, USA) equipped with an electrospray ionization (ESI) source and operated in negative mode. A UPLC separation gradient was developed to efficiently resolve all compounds for a qualitative analysis. The flow rate was set at 0.4 mL/min and the solvent system was: (A) water 0.1% formic acid and (B) acetonitrile. The elution program was: 2% B for 2 min; 100% B in 18 min; and hold for 2 min. After returning to 2% B in 1 min, column equilibration was performed for 4 min at the end of the run. The injection volume was set to 10 μL and the sample was injected at 0.3 mg/mL in water-acetonitrile solution (1:1) on a Supelco Ascentis Express C18 (100 × 2.1 mm i.d, 2.7 μm particle size). The HRMS and HRMS/MS data were acquired in negative mode over 100–1000 m/z range. The MS profile was recorded in full scan mode (scan time = 1 micro scans and maximum inject time = 500 ms). The ESI conditions were as follows: capillary temperature 320 °C; capillary voltage −40 V; tube lens −120 V; ESI voltage 2.7 kV. Nitrogen was used as sheath gas (40 Au) and auxiliary gas (8 Au). Chromatographic and spectrometric features were used for identification of extracts constituents such as retention time (Rt), polarity, accurate m/z, proposed elemental composition (EC), ring double bond equivalent (RDBeq) values as well as HRMS/MS spectra and derived fragmentation motifs. The raw data were acquired and processed with XCalibur 2.2.4 software from Thermo Scientific.

The extract was found to be rich in catechin and epicatechin, phenolic acids, such as gallic acid and caffeic acid and flavonoids (Table 1).

### 2.5. Grape Pomace Extract Administration

GPE was orally administered through drinking water at a dose of 75 mg per day per animal (approximately 280 mg/Kg) for eleven weeks (group C animals) or eight weeks (group D animals).

### 2.6. Behavioral Evaluation

A functional observational battery (FOB; a method used for over 20 years [43,44]) was employed in order to evaluate the oxaliplatin evoked PN and to give an overall characterization of the animals’ general condition (Table 2). FOB is a neurobehavioral assessment tool, widely used in neurotoxicology studies, which consists of several variable tests and was used to investigate the extent and form (motor and/or sensory) of the evoked neuropathy and the possible effects of GPE administration. The FOB tests practiced consist of: i. **home-cage measurements** (body position, respiration rate, palpebral closure), ii. **hand-held observations** (reactivity, handling and palpebral closure upon being held), iii. **open field activity** (gait, arousal, number of rearings, number of fecal boluses and of urine pools, presence of abnormal stereotactic movements, diarrhea), iv. **sensorimotor reflexes** (touch response, sound response and tail pinch response), including responses with exceptional significance such as the righting reflex [45], the contact placing response [46] and the crossed extensor reflex [47], as they are dependent on the corticospinal system and widely used to detect proprioceptive deficits arising from oxaliplatin administration [48], v. **sensorimotor tasks** (footprint analysis, landing foot splay task [49], grip strength task, sticky paper task and Von Frey hair pinch test). For all parameters tested, their validation and scaling refer to Table 2.

The details of the examined sensorimotor reflexes and tasks are: **Righting reflex** describes the animal’s reaction after it has been dropped from a height of 30 cm with its belly up. Normal rats should turn upside down and land on their four limbs, while animals with sensorimotor deficits may land on their side or even on their back. When the dorsal surface of the paw of the hindlimbs of a suspended animal slightly touches the edge of an object, the animal lifts the tested paws and places them on the surface of the object. This reaction is described as the **contact placing response**. Furthermore, the **crossed extensor reflex** is elicited when in a suspended animal the one hind limb is pinched; as a response, the irritated hind limb is flexed, and the contralateral hind limb is extended.

**Landing foot splay** is the measured distance between the imprints of the heels of the two hind limbs, validated after dropping the animal from 30 cm high on to a surface, covered with white recording paper. Several studies prove that animals with sensorimotor deficits are present with increased hind limb spread in the landing foot splay task [49].

**Grip strength** can accurately demonstrate sensorimotor peripheral neuropathy [50]. It is a reliable indicator of touch and proprioceptive sensation as a function of motor efferent fibers and muscle use [51]. Animals were forced to grab a wooden bar, linked to an appropriate dynamometer (Slim, Pen Scale) with their hind limbs and the maximal strength, seen at the time that their hind limbs are being released from the apparatus, was recorded.

The **sticky paper test** was applied to evaluate the animal’s reaction to light touch stimuli. A self-adhesive paper (14 × 23 mm) was placed onto the paw of the right hind limb and the latency of the rat’s first reaction (paw licking, lifting or removal) was recorded at each study point. There was a cut off time of 120 s.

The **Von Frey hair pinch test** is widely used to examine “pain-like” behavior including changes in mechanical thresholds in rodents [52]. In this test, animals were placed individually in small cages with a penetrable bottom. Von Frey hair filaments of increasing grams and diameter were applied perpendicularly to the external tibial surface of all animals and the filament that succeeded to elicit a positive touch response (paw withdrawal, licking or shaking) was recorded. The “ascending stimulus” method was used [53] beginning the assessment to the response to a filament of the lowest force (in this case 0.4 g-force) for a set number of applications (in this case five times). If the response rate is less than 40% (i.e., a withdrawal response is elicited in none or one out of five applications) the next filament is tested. If the response rate is 40% or more (i.e., withdrawal response is elicited in two or more out of five applications) testing stops and the force of the last von Frey filament is designated as the mechanical withdrawal threshold (in this case 1,5 g-force).

**Figure 2:**
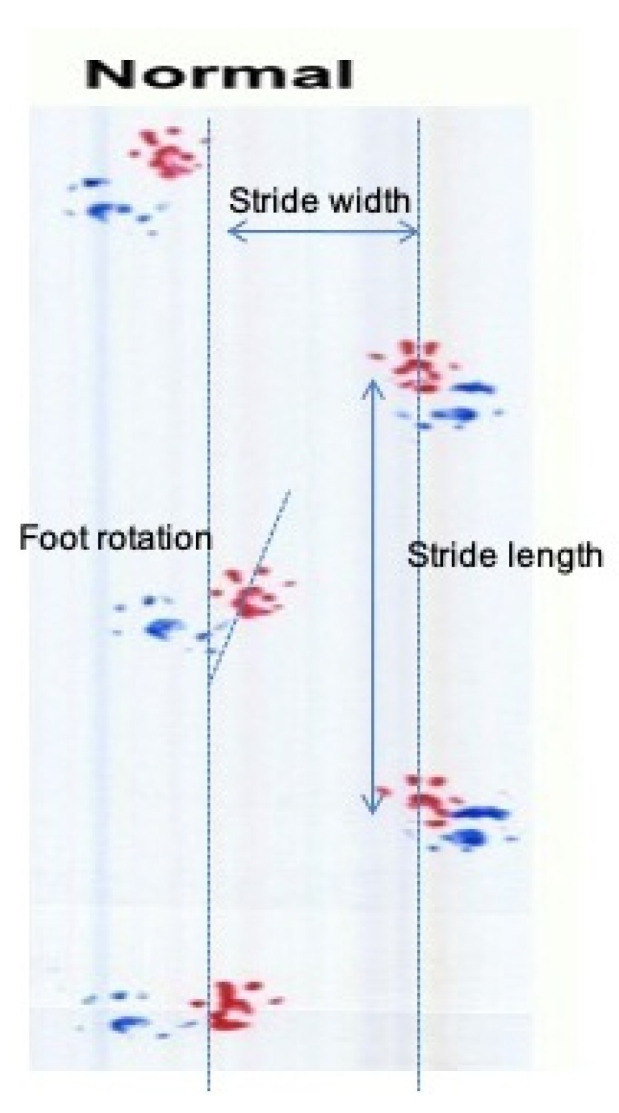
Photograph of the white paper showing marks of the hind (blue) and front (red) paws of five sequential steps for the evaluation of animal’s stride width, stride length and rotation of the right and left hind limb. Interpedal distance was calculated as described in the text.

For the gait (footprint) analysis, rats were forced to walk on an 80 cm long and 10 cm wide runway covered with white paper after having their hind paws inked with non-toxic colors. Marks of the hind paws of five sequential steps were used to evaluate animal’s stride width, stride length, interpedal distance and rotation of the right and left hind limb (Figure 2). Interpedal distance is the mean of five measurements of the distance between the 1st right and the contralateral (1st left) foot, the 1st left with the 2nd right and so on, for the three steps depicted in the Figure 2.

Tests were performed in all animals at four different time points; prior to any treatment, at the middle of the oxaliplatin treatment period, at the end of the oxaliplatin treatment period and finally at three weeks after the treatment period. The landing foot splay, grip strength and sticky paper tests were performed three times at each tested period and the average score was presented. Tests were practiced at three sequential days each time with a time interval of 30 min between the tests, blindly by the same persons each time.

An extensive description of the FOB tests used and the scoring scale used to express the data have been published elsewhere [54,55].

### 2.7. Blood Sample Collection and Oxidative Stress Biomarkers Evaluation

At the end of experimentation, animals were injected i.p. with a ketamine/xylazine mixture (50 and 5 mg/Kg, respectively). Blood samples were collected from all animals to estimate total antioxidant capacity (TAC) values and to perform the thiobarbituric acid-reactive substances (TBARS) assay. Blood was collected after cardiac puncture and was properly centrifuged (1370× *g* for 10 min at 4 °C). Acquired plasma samples (centrifugation’s supernatant) were collected and stored at −80 °C until used.

Total antioxidant capacity determination was based on the method of Janaszewska and Bartosz [56]. Briefly, 20 μL of plasma, 480 μL of 10 mM sodium potassium phosphate (pH 7.4) and 500 μL of 0.1 mM DPPH free radical were mixed and incubated in the dark for 60 min at room temperature. Samples were centrifuged for 3 min at 15,000× *g*, and the optical density was measured at 520 nm. TAC is presented as mmol of DPPH reduced to 2,2-diphenyl-1-picrylhydrazine (DPPH:H) by antioxidants of plasma.

Thiobarbituric acid-reactive substances assay was used for the determination of lipid peroxidation. TBARS were determined according to a slightly modified assay of Keles et al. (2001) [57]. Specifically, 100 μL plasma, 500 μL of 35% TCA and 500 μL of Tris–HCl (pH 7.4) were mixed and incubated for 10 min at room temperature. Then, one milliliter of 2 M Na2SO4 and 55 mmol/l thiobarbituric acid solution was added, and the samples were incubated at 95 °C for 45 min. The samples were cooled on ice for 5 min, and subsequently vortexed after the addition of 1 mL of 70% TCA. The samples were centrifuged at 15,000× *g* for 3 min, and the optical density of the supernatant was determined at 530 nm. TBARS are expressed in terms of malondialdehyde (MDA) equivalents. The molar coefficient of MDA is 155 × 103 mol/L.

### 2.8. Tissue Preparation and Immunohistochemistry

Following blood collection, all animals were perfused with 100 mL of 0.9% sodium chloride followed by 300 mL of 4% paraformaldehyde. Sensory neurons that innervate rat’s hind limbs, clinically evaluated for the presence of PN, are located at L3 to L6 lumbar spinal cord level [58], and so the right and left fourth, fifth and sixth lumbar dorsal root ganglion (DRG) of all animals were collected and fixed in 4% paraformaldehyde overnight. Then tissues were processed routinely and embedded in paraffin. The right fourth lumbar DRG of all experimental animals was sectioned perpendicular to its longitudinal axis at 8 μm and treated either for haematoxylin and eosin (H&E) or immunofluorescence staining.

To assess certain morphometrical and histological characteristics of untreated and oxaliplatin and/or GPE treated DRG sensory neurons, one of ten serial sections of each DRG was stained with H&E. An average of ten sections per animal were stained and evaluated afterwards.

Double immunofluorescence staining against parvalbumin and Glial Fibrillary Acidic Protein (GFAP) was performed in order to evaluate the presence of large DRG sensory neurons along with the satellite cells excitation in response to evoked pathology [21,59,60]. Antibodies raised against mouse parvalbumin (1:1.000, Sigma, St. Louis, MO, USA) and rabbit GFAP (1:500 Dako/Agilent, Santa Clara, CA, USA) proteins were used. Selected sections were first deparaffinized and dehydrated through a series of xylene and alcohol solutions. After antigen retrieval pretreatment in Citrate Buffer (PH = 6) under heating, sections were incubated with appropriate blocking serum solution (5% Normal Goat Serum, 1% Triton-X in PBS) in RT for 2 h. Primary antibodies were applied overnight at 40 C and then tissues were incubated for 1 h at RT with appropriate secondary antibodies (1:200, mouse IgG or 1:200 rabbit IgG, both from Biotium, Hayward, CA, USA) and covered with fluorescence mounting medium (DAKO, Denmark).

### 2.9. Morphometrical Analysis

Hematoxylin-eosin (H&E) stained sections were examined under a Nikon optical microscope (Nikon Eclipse 80i microscope; Nikon Europe B.V., Amsterdam, Netherlands) equipped with a Nikon D-Eclipse C1 camera. Morphometrical analysis data were obtained with the Image Analysis Pro-Plus 6.3 Program for Windows (media Cybernetics, Rockville, MD, USA). Certain morphometrical parameters of DRGs of different experimental groups such as the mean cell area, mean diameter and perimeter and the ratio of small (<500 μm^2^), medium (500 μm^2^–1000 μm^2^)and large (>1000 μm^2^) DRG sensory neurons [61] were evaluated. All above parameters, and especially the percentage of large DRG cells, could be somewhat underestimated compared to similar studies using cryostat sections, due to the shrinkage of the tissues following dehydration during their histological processing.

In order to estimate total DRG neuron cell number, the number of neurons with nucleolus present in every tenth cut of each ganglion was measured and the sum of these measurements was multiplied by the number 10 [62]. It has been shown, however, that often DRG neuron cells are present with two nucleolus and so the use of a correction factor (c.f.) was necessary. To calculate c.f., in a random area of a random cut of each ganglion fifty neurons with visible nucleolus were measured (n_c.f._ =50). Then the previous and the next cuts were checked to see if measured neurons were present synchronously and with a second nucleolus and the total nucleolus number (N_c.f._) of these fifty neurons was recorded. Then c.f. was calculated as follows: c.f.= N_c.f._/n_c.f._, and was multiplied with the total DRG neuron number, found after measuring neurons in every tenth cut, giving us the final total number of the DRG neurons.

Immunofluorescence sections were examined with a Nikon D-eclipse C1 confocal laser scanning microscope (CLSM) and image recordings were captured with appropriate software (EZ-C1 3.20) and presented as z-Stacks. Laser beam gain was set at a specific point, which was stable for all studied sections of different experimental groups.

### 2.10. Statistical Analysis

All data were analyzed using the SPSS 23.0 statistical software. Behavioral testing parameters were ranked (according to a defined scale), measured or validated as present/absent. Ranked and descriptive data were analyzed using a Chi-square test. Measured FOB variables, body weight measurements and oxidative stress marker score were examined using a one-way ANOVA (Tukey’s post hoc analysis) and independent samples t-test were used for comparisons between groups. Homogeneity of variances was tested using the Levene’s test and when violated, the non-parametric two-tailed Kruskal–Wallis was assessed for multiple comparisons, followed by the Mann–Whitney U test (two-tailed) for two-by-two comparisons. All measured data are expressed as mean ± S.E.M. Significance was set at *p* < 0.05.

## 3. Results

### 3.1. Oxaliplatin and GPE Administration Effect on General Appearance, Locomotion and Exploratory Activity of Experimental Animals

During the experimental period, no fatigue, abdominal bloating, alopecia or kyphosis of treated animals were observed at any studied time point. As revealed by home-cage measurements, no statistically significant differences at body position, respiration rate and palpebral closure were found between experimental groups. Furthermore, reactivity, handling and palpebral closure of tested animals were found to be normal at all oxaliplatin and GPE treated rats.

Open field test is widely used to measure locomotor activity and anxiety-like behavior of an animal when placed in a novel environment [63,64]. Gait and arousal of all studied animals were found to be normal at all experimental time points. No significant differences in exploratory activity, measured by number of rearings (group A, 6.85 ± 1.32; group B, 9.2 ± 1.32; group C, 6.86 ± 1.08; group D, 6.6 ± 1.1), and in activity of the autonomic nervous system, represented with number of defecations (group A, 1.050 ± 0.29; group B, 0.9 ± 0.29; group C, 1.23 ± 0.24; group D, 0.95 ± 0.24), were found between control and experimental groups.

### 3.2. Oxaliplatin Administration and GPE Consumption Have No Effect on Body Weight

Body weight of experimental animals was recorded twice a week, on drug or dextrose administration days, and data are presented in Table 3. No statistically significant differences in mean body weights were found to exist at any examined treatment period among the different experimental groups.

### 3.3. Animals Receiving GPE Show NORMAL Sensorimotor Reflexes Whereas in Animals Receiving Only Oxaliplatin Certain Reflexes Were Severely Harmed

All experimental animals were present with normal approach and sound response at all studied time points. A normal eyelid and righting reflex were also evident for all animals. There were some differences observed in the following tests:

**Tail pinch response (TPR):** while at the start of experimentation all tested animals were present with a normal response, at the end of oxaliplatin treatment 60%, 16.67% and 22.22% of group B, C and D animals, respectively, showed no reaction after having the base of their tail squeezed with tweezers. Statistical evaluation revealed that group B values differed significantly (*p* ≤ 0.05) from control values, whereas no statistical difference between control and group C and D responses was found. Interestingly, following the three weeks after-treatment period, all group C and D animals were present with a normal response, whereas 20% of group B animals were still lacking the tail pinch response.

**Contact placing response (CPR):** With the use of this reflex, severely affected proprioception by oxaliplatin administration, can be clinically evaluated. Just from the middle of oxaliplatin treatment, 60% of group B animals had a normal reflex in only one hind limb, showing a statistically significant difference from controls (*p* ≤ 0.05), group C (*p* ≤ 0.05) and group D (*p* ≤ 0.1) where only 12.5% and 11.11%, respectively, of treated animals showed a decreased response. At the end of the after-treatment period, all group C and D animals had a normal score, while group B animals significantly differed (*p* ≤ 0.05) as 40% were still present with a decreased response.

**Crossed extensor reflex (CER):** Amazingly, at the end of the oxaliplatin treatment, all group B animals showed no crossed extensor reflex, whereas 55.56% of group D animals were present with a normal reflex, and this difference was considered as statistically significant (*p* ≤ 0.05). A recovery was seen at the end of the after-treatment period, with 40% of group B and all group D animals having normal reflexes.

In the latter three tested reflexes, an oxaliplatin induced significant decrease was evident. Groups receiving GPE had a better score in these three tested reflexes at all studied time points and also showed an increased repairing ability in the post-treatment period. All above data suggest that GPE has a prophylactic effect on the nervous system.

### 3.4. GPE Administration Has Not Altered Gait Analysis Measurements of Oxaliplatin Treated Animals

As mentioned earlier we evaluated animal’s stride width, stride length, interpedal distance and rotation of the right and left hind limb (Figure 2) during the gait analysis test. Below are the results from these measurements (Table 4).

**Foot rotation**: All oxaliplatin treated groups were present with an increased rotation of the R and L hind limb compared to control animals, although this was not evaluated as statistically significant. Foot rotation is indicative of the sciatic nerve function, possibly affected by oxaliplatin administration.

**Stride length:** Although there are some slight differences between the animal groups regarding the stride length of both feet, there is no statistical significance between them. This leads to the observation that ataxia, coordination and locomotion, which are mirrored into the stride length, have not been altered.

**Stride width:** All oxaliplatin treated groups showed decreased stride width values. The observed difference was not found to be of statistical significance. Thus, the locomotion and the base of support, two characteristics implied by the measurement of the stride width, despite showing a decreasing tendency among the untreated and the oxaliplatin treated animals, is not indicative of motor neuropathy.

**Interpedal distance:** as in previous measurements, there are some slight differences between the animal groups regarding the interpedal distance. No statistical significance was evident between them.

### 3.5. Effect of GPE Administration on Sensorimotor Tasks

Along with gait analysis, four more sensorimotor tasks were performed on all animals and gave us an informative overall characterization of the altered neuromuscular function evoked by oxaliplatin administration. Animals receiving GPE along with oxaliplatin treatment were present with mean task values closer to the untreated control values, whereas in group B animals’ severity of motor and sensory impairments was greater. As expected, the measured distance during the **landing foot splay** task between the imprints of the hind feet was increased in group B, C and D animals (4.35 ± 0.34 cm, 4.43 ± 0.29 cm and 4.58 ± 0.28 cm, respectively) compared to untreated control animals where measured distance was 3.99 ± 0.5 cm. However, no statistically significant differences among oxaliplatin treated groups exists.

Significant alterations in measured **hind limb force** between tested animals were also evident. A statistically significant difference in hind limb muscle strength between group B and group C (*p* ≤ 0.05) and D (*p* ≤ 0.01) animals was indicated. Group C and D animals were present with increased grip strength values (3.398 ± 0.146 N and 3.529 ± 0.147 N, respectively) whereas group B animals showed exceptionally reduced values (2.807 ± 0.178 N).

Affected nociceptive ability of oxaliplatin treated animals was revealed with the use of the **sticky paper test**. At the end of oxaliplatin treatment, mean latency of control animals was 157.68 ± 23.98 sec whereas group B and C animals showed a quicker reaction at 124.92 ± 24.31 and 126.77 ± 18.99 sec, respectively (Table 5). Although it is evident that group B and C oxaliplatin treated animals showed a reduced reaction time compared to controls this was not evaluated as statistically significant due to the large variability of values. Group D animals reacted to the stimuli within 161.07 ± 18.51 sec, close to the controls’ reaction time. The employed test is indicative of the touch receptors and motor efferent fibers function of the animals tested. Hence, oxaliplatin might have possibly caused hyperalgesia, which was partially reversed by the GPE administration.

**Figure 3:**
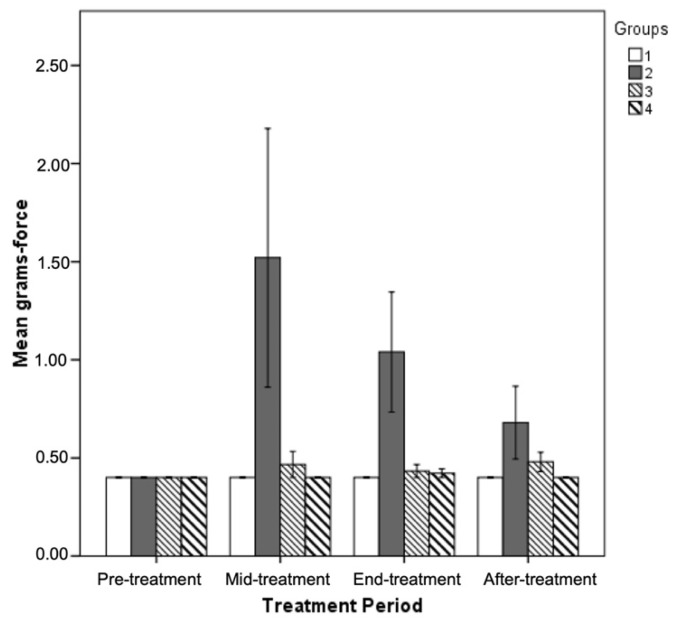
Mean grams of applied Von Frey hair filaments that elicited a positive touch response of the hind limb in experimental rats during all examined time periods (Group A = 1; Group B = 2; Group C = 3; Group D = 4).

**Von Frey hair** filaments of increasing grams and diameter were applied to the hind limbs of experimental animals to determine animals’ mechanical threshold. At the end of oxaliplatin treatment period, group B animals were presented with a significantly increased mechanical threshold as compared to control and group C and D animals (Table 5) and this pattern was also observed at all examined time points (Figure 3). However, the mechanical threshold of group B animals tends to decrease after the middle-treatment period. On the contrary, thresholds of group C and D animals did not differ significantly from thresholds of control animals at any examined time point.

### 3.6. Oxidative Stress Biomarkers Evaluation

TAC and TBARS levels were performed into plasma samples of animals belonging to all experimental groups.

The evaluation of oxidative stress biomarkers (Table 6) revealed a proposed statistically significant decrease in TAC levels in the simultaneous treatment of oxaliplatin and GPE group (group D) compared with the oxaliplatin group (group B). TBARS levels did not exert any statistically significant difference. Our data clearly designate that GPE induced a prooxidant effect in the experimental group D, when administered in combination with oxaliplatin treatment. Nevertheless, the prior administration of GPE and the subsequent treatment with oxaliplatin (group C) did not induce any significant alteration in the redox status of the animals.

### 3.7. Oxaliplatin or GPE Administration Have No Effect on Total Number of Lumbar DRG Sensory Neurons

Our data clearly demonstrate that total lumbar DRG neuron cell number did not differ between control and oxaliplatin treated rats (Table 7). Furthermore, sensory neuron number was unaffected by GPE administration, even when counted populations of oxaliplatin and oxaliplatin/GPE treated groups were almost equal. So, it can be excluded that the clinically observed neuroprotective effect of GPE is not due to possible inhibition of DRG cell death or apoptosis driving decreased neuronal populations.

### 3.8. GPE Protects Large Lumbar DRG Sensory Neurons from Oxaliplatin Induced Atrophy

**Figure 4:**
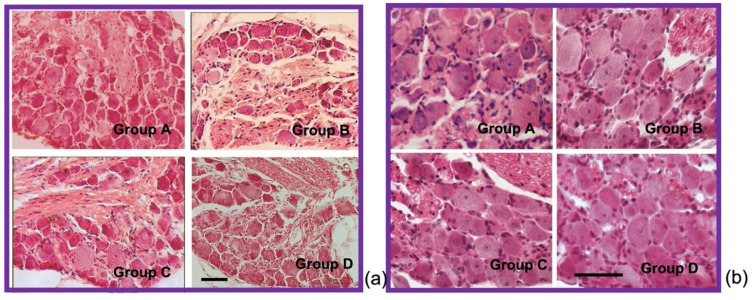
Microphotographs of experimental groups’ DRGs. Note the significant difference in mean DRG neurons somatic area between group B and group C and D animals (**a**) ×20 and the increased satellite cells infiltration in group B DRGs compared to group C and D (**b**) ×40. (scale bar = 50 μm).

Large lumbar DRG sensory neurons are proven to be primary oxaliplatin drug targets, thus leading to their atrophy and to the substantial reduction in mean somatic area of DRG sensory neurons. So, along with the estimation of total DRG neuronal cell population of experimental groups, the percentages of small (≤500 μm^2^), medium (500–1000 μm^2^) and large (≥1000 μm^2^) DRG sensory neurons were estimated. The ratio of the large DRG cell population was found to be greater in group C and D compared to group B animals (Table 7, Figure 4a). No significant differences in ratio of large DRG neurons between group C and D were found, suggesting the neuroprotective effect of GPE towards large sensory neuron induced atrophy is irrelevant of the time of GPE administration.

Higher large DRG cell proportions in group C and D animals lead to increased values of mean somatic area as opposed to group B animals. Mean somatic area of DRG sensory neurons was higher in group C (393.92 ± 3.39 μm^2^) and D (428.87 ± 2.82 μm^2^) compared to group B, where mean area was found to be decreased at 390.94 ± 2.48 μm^2^.

### 3.9. Histological and Immunofluorescent Staining Revealed Increase in the Satellite GFAP-Stained Cells in the Oxaliplatin Alone Treated Animals

**Figure 5:**
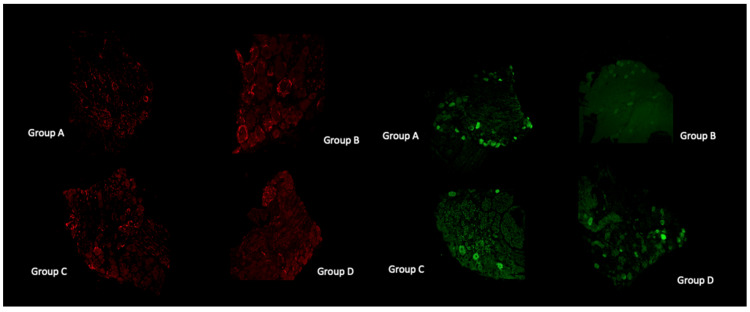
Microphotographs of experimental groups’ DRGs. On the **left**, the images are showing GFAP, while the **right** panel shows parvalbumin immunohistochemistry staining (×10).

**Figure 6:**
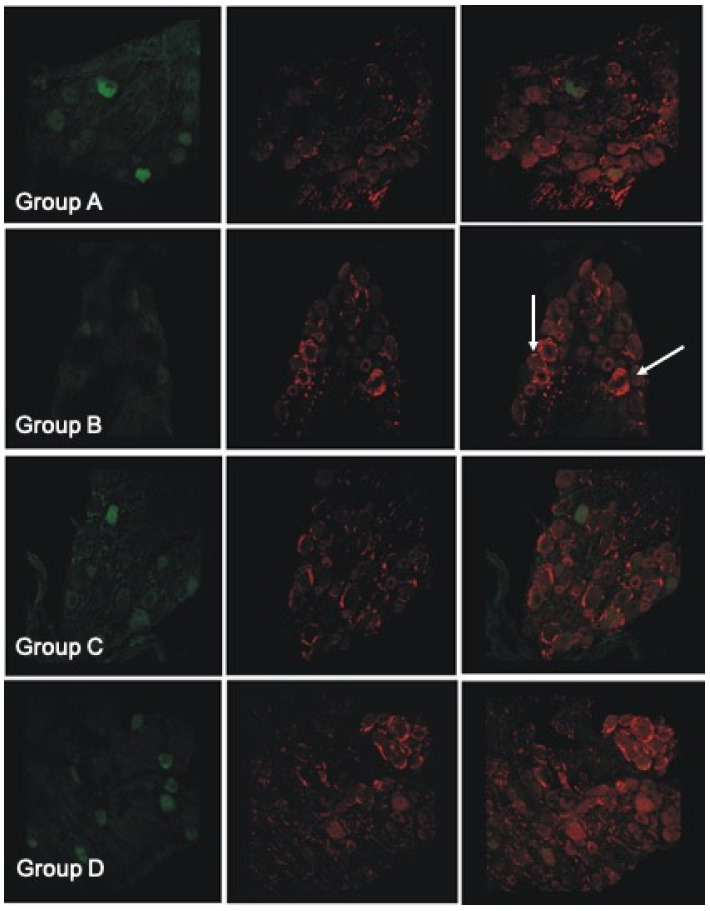
Microphotographs showing double (merged) staining of parvalbumin (green) and GFAP (red) immunohistochemistry staining (×20) in all experimental groups. Note the heavily GFAP stained satellite cells (white arrows) for the oxaliplatin treated (Group B) animals.

Observation under light microscopy of pathological DRGs from all three oxaliplatin treated groups, revealed both a significant difference in the mean somatic area of the DRG neurons, and an exceptionally increased satellite cell infiltration in group B lumbar DRGs compared to GPE treated (Figure 4b). Satellite cells increased in number and size and encircle the sensory DRG neurons, as revealed by GFAP staining (Figure 5 (left panel) and Figure 6).

Merging of parvalbumin (green) and GFAP (red) immunohistochemistry staining in all experimental groups (Figure 6) revealed the encircling of large DRGs with GFAP stained satellite cells in the oxaliplatin treated group. The number of large (parvalbumin stained) DRG cells is diminished in the oxaliplatin treated (group B) animals. Instead, increased GFAP staining of the satellite cells was evident (white arrows). In group C and D images, parvalbumin stained (green) cells colocalize with GFAP (red) staining less when compared with group B.

## 4. Discussion

Oxaliplatin has been widely used over the last few decades for the therapy of many malignant manifestations, such as colorectal, ovarian, lung, prostate and pancreatic cancer [65]. Despite its advantages over other platines used as therapeutic regimes for the same dilapidating diseases, oxaliplatin is inducing two types of peripheral neuropathies (PN) as side effects [66]. The first observed acute side effect is resolved quickly after its appearance, but the chronic one stays for an unbalanced time period leading repeatedly to the discontinuation of the therapy [16].

Many attempts are being made by researchers to overcome this obstacle of the very beneficial oxaliplatin. Among the substances evaluated for neuroprotection from the oxaliplatin induced chronic neuropathy are anti-oxidants, since it is well known that platinum compounds, such as oxaliplatin, induce oxidative stress through mechanisms involving nuclear and mitochondrial DNA dysfunction, as well as mitochondrial oxidative damage and depletion of non-enzymatic antioxidants [32,67].

Grape pomace extract is rich in antioxidant molecules. It possesses high quantities of catechins, epicatechin, procyanidins, phenolic acids (mainly gallic and caffeic acid) and flavonoids (Table 1) that are known for their ability to prevent chemotherapy-induced oxidative cell and mitochondrial damage. Catechins induce heme oxygenase-1 expression, through activation of the Nrf2 transcription factor, and in this way protecting neurons from oxidative stress-induced cell death [68]. Grape seed procyanidins were also shown to rescue renal cells from cisplatin-induced damage by a similar pathway [69]. In a recent study, gallic acid showed a protective effect against cisplatin-induced mitochondrial oxidative stress, by decreasing mitochondrial ROS formation, membrane damage and malondialdehyde (MDA) and increasing mitochondrial glutathione (GSH), superoxide dismutase (SOD), glutathione peroxidase (GPX) and catalase [70].

In our study, we used a grape extract obtained from pomace in oxaliplatin treated rats to investigate its possible advantageous effect on oxaliplatin induced PN. Along with the exploration of TAC activity into the blood of all animals, we applied different behavioral tests to estimate the produced neuropathy and used histology and immunohistochemistry to evaluate the morphometric data of the affected neurons.

To our knowledge, this is the first time that the FOB assessment tool is used to explore oxaliplatin induced chronic neuropathy in rats.

### 4.1. Oxaliplatin and GPE Administration Effect on General Appearance, Locomotion and Exploratory Activity of Experimental Animals

The first tests of the FOB neurobehavioral assessment tool that we practiced on the animals, consisted of home-cage measurements, hand-held observations and the open field activity (Table 2). We observed no fatigue, abdominal bloating, alopecia or kyphosis in the animals. In some cases, ascites was a post-mortem finding and it did not affect the general appearance of the animals. No differences were detected in home-cage and hand-held observations or in their open field test parameters. This observation implies natural exploratory and autonomic nervous system activity (defecation and urination numbers) in all groups. The anticipated results from these tests are in accordance with the work of Jamieson et al., 2005 [21] and Sakurai et al., 2009 [65].

### 4.2. Oxaliplatin Administration and GPE Consumption Have No Effect on Body Weight

Body weight of experimental animals was recorded twice a week, on drug or dextrose administration days. No statistically significant differences in mean body weights were found to exist at any examined treatment period among the different experimental groups (Table 3). Animals in all four groups gained weight (although it was not statistically significant) after oxaliplatin and GPE administration ended, resulting in an increase in their mean body weight at the end of the post-treatment period. This fact can be explained by the inevitable stress produced by the handling and the injections. Most studies did not detect any significant decrease in the body weight of the animals used [65,71], although body weight was significantly reduced following treatment with oxaliplatin [21]. This discrepancy can be attributed to the dose concentration and/or duration of oxaliplatin treatment along with the differences in sex, strain and rodent species used in each project [72].

### 4.3. GPE Rescues the Oxaliplatin-Induced Damage on Corticospinal Funtion

The functional observational battery (FOB) is a noninvasive procedure designed to detect sensorimotor and gross functional deficits in young adult rats following their exposure to chemicals and to better quantify the evoked neurotoxic effects _71._ In our study we speculated the usefulness of FOB to understanding the type of toxicity induced by oxaliplatin and to evaluate the sensorimotor benefits of GPE administration.

All oxaliplatin-receiving animals presented a normal approach and sound response at all studied time points. Normal eyelid and righting reflexes were also evident for all animals (Table 2). On the other hand, oxaliplatin induced a significant decrease in the tested reflexes involving the corticospinal function. More specifically, regarding the **crossed extensor reflex**, all oxaliplatin treated animals felt the pain and withdrew the infected hindlimb, possibly due to the evoked paresthesia (increased sensitivity of nociceptors). On the contrary, they did not extend the contralateral hindlimb, which may be due to the histological damage that did not allow the stimulus to go on the contralateral side of the spinal cord.

Proprioception of experimental animals was also tested through the **contact placing response** and oxaliplatin treatment was shown to significantly hamper proprioception of treated animal. This may be due to damage in large DRG neurons, which is evident in the morphometrical analysis of the DGR neurons, in accordance with numerous other publications [18,21,73,74].

GPE treated animals presented higher percentages of normal proprioception at all studied periods. Results from examined responses and reflexes reflect the oxaliplatin-induced damage, mainly to corticospinal reflexes, and the prophylactic effect of the GPE.

### 4.4. Oxaliplatin and GPE Administration Has Not Altered Gait Analysis Measurements of Oxaliplatin Treated Animals

Following the reflexes, we tested the gait of the experimental animals and we evaluated animal’s stride width, stride length, interpedal distance (the mean of five measurements) and rotation of the right and left hind limb (Figure 2).

All the observed differences were found to be without statistical significance (Table 4), evidencing the intact gait characteristics of all experimental animals. This follows the evidence that oxaliplatin induced neuropathy is mainly sensory and not motor. Gait difficulties are also not typically found in oxaliplatin-receiving individuals, where, the observed motor symptoms in humans include tetanic spasms, fasciculations and/or prolonged muscular contractions [11,75].

### 4.5. Beneficial Effect of GPE Administration on Sensorimotor Tasks

In order to better quantify the oxaliplatin-evoked damage in sensorimotor tasks and to test the possible benefits from GPE administration, we also performed the landing foot splay task, the grip strength task, the sticky paper task and the Von Frey hair pinch test.

All oxaliplatin treated animals presented increased **landing foot splay task** values compared to untreated control animals and GPE administration did not exert any beneficial effect (Table 5).

When examining the **hind limb force** of animals, all three oxaliplatin treated groups showed a gradually decreased **hind limb force** during the oxaliplatin treatment period. However, GPE-receiving animals presented significantly higher values of grip strength measurements at the end of oxaliplatin treatment period (Table 5). In a recent publication by Lees et al. [76] muscle wastage of oxaliplatin treated individuals was considered to be the reason for the non-beneficial effect of exercise on oxaliplatin driven neuropathy. Muscle waste might be the reason for our observations of reduced grip strength noticed in the oxaliplatin alone group.

Regarding the **sticky paper test** (Table 5), oxaliplatin treated animals showed a reduced reaction time, although not significant. Group D animals reacted to the stimuli close to controls’ reaction time. The employed test is indicative of the function of the touch receptors and of motor efferent fibers’ function of the animals tested. Hence, oxaliplatin might possibly have caused mechanical allodynia, which was partially reversed by the GPE administration. The mammalian low threshold mechanoreceptors are Aβ fast conducting nerve fibers (ranging from [30 to 100 m/s], averaging ~40–60 m/s) have large soma size/axon diameters and are highly myelinated [77]. The reduced reaction time of the oxaliplatin treated animals follows our morphometric values for DRG neurons where large DRG cells were strongly affected by the platinum-treatment. Besides, mechanical allodynia has been reported in numerous rodent models of platinum induced chronic neuropathy. For a review see Kawashiri et al. (2021) [13]

**Von Frey hair** filaments of increasing grams and diameter were applied to the hind limbs of experimental animals to determine their mechanical threshold. The smaller diameter von Frey filaments that we employed, are measuring “pain-like” activity transported through Aδ and C fibers. Our results showed that the oxaliplatin treated animals responded to 1.5 gr testing of von Frey filaments indicative of a decreased sensation of pain and/or light touch. The noticed reduced sensitivity was reversed following the GPE administration (for the group C and D animals; Table 5, Figure 3). Although a mechanical hyperalgesia of oxaliplatin treated animals was anticipated from previous reports on oxaliplatin induced rodent models [78], care should be taken in the interpretation of the results depending on the dose, route of administration, schedule of oxaliplatin treatment in combination with the von Frey filaments measured in each study. Following are some examples of the abovementioned observation: in the work of Sakurai et al. (2009) [65] oxaliplatin (1, 2 and 4 mg/kg) was administered i.p. twice a week for 4 weeks. Boyette-Davis et al. (2011), [78] used oxaliplatin (Tocris) diluted to a concentration of 1 mg/mL using saline and given at a dosage of 2 mg/kg every other day for a total of four injections (Days 1, 3, 5 and 7). Ahn et al. (2014) [79] evaluated mechanical hypersensitivity by a tail immersion test in cold water (4 °C) and a von Frey hair test. These authors used oxaliplatin (Sigma, Ronkonkoma, NY, USA), dissolved in a 5% glucose (Sigma, Ronkonkoma, NY, USA) solution at a concentration of 2 mg/mL, and it was intraperitoneally administered at 6 mg/kg. Testing was initiated with a hair, of which the bending force was 2.0 g. In conclusion, in our opinion, this is a subject that needs more clarification.

Largely, other works studying the features of the peripheral neurotoxicity observed following the use of the platinum-based drugs with neurophysiological, pathological and analytical methods in several well-characterized animal models [17] noticed a decrease in nerve conduction velocity induced by damage to neuronal cell bodies and peripheral axonopathy, which is observed in our animal model as well.

### 4.6. GPE Effect on the Oxidative STRESS Induced by Oxaliplatin

As mentioned earlier in the text, oxidative stress is a possible factor for the chronic neuropathy induced by different platines. Hence, research focuses on different antioxidant substances that might be beneficial for this oxaliplatin induced side effect. So far, the antioxidant compound silibinin [26], the potential of phosphatidylcholine (PC) in rats [80], the effects of the ethanolic extract of X. aethiopica (XAE) and its diterpene xylopic acid (XA) in vincristine-induced neuropathic pain have been tested [81]. In this list of possible antioxidants for the neuroprotection of oxaliplatin, induced neuropathy GPE can be added.

In a previous study, a grape leave hydroalcoholic extract prevented oxaliplatin-induced oxidative damage in primary astrocytic cultures and reduced the TBARs values [40]. In our experiments, TBARs values in the blood of treated animals were unaffected of oxaliplatin and/or GPE treatment. However, a significant decrease in TAC levels in the animals that simultaneously received oxaliplatin and GPE group (group D), compared with the oxaliplatin group (group B) was observed. It seems that GPE may induce a prooxidant effect in the experimental group D, when administered in combination with oxaliplatin treatment, whereas the prior administration of GPE and the subsequent treatment with oxaliplatin (group C) did not induce any significant alteration in the redox status of the animals.

### 4.7. Oxaliplatin or GPE Administration Have No Effect on Total Number of Lumbar DRG Sensory Neurons

As already assessed by others, oxaliplatin did not cause neuronal death or apoptosis of DRG cells [21]. In our study, the total lumbar DRG neuron cell number did not differ between control and oxaliplatin treated rats (Table 7) and the sensory neuron number was unaffected by GPE administration. So, it can be concluded that the clinically observed neuroprotective effect of GPE is not due to possible inhibition of DRG cell death or apoptosis driving to decreased neuronal populations.

### 4.8. GPE Protects Large Lumbar DRG Sensory Neurons from Oxaliplatin Induced Atrophy

Our results on morphometrical analysis of DRG neurons suggest a neuroprotective effect of GPE towards large sensory neuron induced atrophy, which is irrelevant of the time of GPE administration (Table 7). This finding is supported by the parvalbumin immunofluorescence staining (Figure 4), showing diminished staining in the oxaliplatin treated group. The rest of the animal groups presented with more parvalbumin stained DRG cells compared to the oxaliplatin treated animals. Our results are in accordance with previous studies [18,19,21].

### 4.9. Histological and Immunofluorescent Staining Revealed Increase in the Satellite GFAP-Stained Cells in the Oxaliplatin Alone Treated Animals

Observation under light microscopy of pathological DRGs from all three oxaliplatin treated groups, revealed both a significant difference in the mean somatic area of the DRG neurons, and an exceptionally increased satellite cell infiltration in group B lumbar DRGs compared to GPE treated (Figure 4b). Satellite cells increased in number and size and encircle the sensory DRG neurons, as revealed by GFAP staining (Figure 5 and Figure 6). This has also been evidenced in many other preclinical studies of platinum evoked PN [82]. In the study of Micheli et al., 2018 [40] repeated oxaliplatin treatment also resulted in a numeric increase in GFAP-positive astrocytes in the spinal cord. Administration of *Vitis Vinifera* GPE along with oxaliplatin treatment significantly decreased satellite cells infiltration of the DRGs.

The solely oxaliplatin group was presented with decreased large DRG cells, which is in accordance with other publications, since large DRG cells are affected by oxaliplatin. The same group (group B) showed GFAP in abundancy. GFAP staining encircled the large DRG, which is indicative of a long-lasting increase in oxidative stressors in the DRGs due to oxaliplatin. Abundance of satellite cells is generally indicative of inflammation in the region (e.g., monoarthritis in rat) [83]. Xu et al., 2021 [84] did not notice GFAP co-localization with the NOX2 (which is an oxidation producer) following neuropathy produced by nerve injury. Goncalves et al., 2018 [32] evidenced upregulation of satellite glial cells following diabetic neuropathy. The same was also noticed in rodents following streptozotocin treatment [85]. Besides that, it is known from earlier works that GFAP has increased expression in DRGs from axotomized rat sciatic nerve [86] where it was hypothesized that GFAP stained cells elevation was due to changes in satellite shape or motility.

## 5. Conclusions

In conclusion, this study has shown the beneficial effect of a grape pomace extract, as an antioxidant regimen due to its compounds’ antioxidant effects to an oxaliplatin induced chronic neuropathic rat model. Rodent models have played an important role in the confrontation of the undesirable effect of oxaliplatin. Strain, sex and dose differences should be evaluated in the future for an integrated therapy of the chronic neuropathy.

## Figures and Tables

**Table 1:** (−) LC-HRMS of the grape extract.

	Rt (min)	Compounds	Theoretical [M-H]^−^ *m*/*z*	Experimental [M-H]^−^ *m*/*z*	Molecular Formula	RDBeq.	Delta (ppm)
1	0.75	Gluconic acid	195.0510	195.0511	C_6_H_12_O_7_	1.5	0.50
2	1.10	Gallic acid	169.0142	169.0145	C_7_H_6_O_5_	5.5	1.32
3	1.90	Protocatechuic acid	153.0193	153.0195	C_7_H_6_O_4_	5.5	1.29
4	2.17	Dihydroxybenzoic acid hexoside isomer 1	315.0722	315.0724	C_13_H_16_O_9_	6.5	0.84
5	3.01	Procyanidin B1 or B2	577.1351	577.135	C_30_H_26_O_12_	18.5	−0.20
6	3.03/3.34	Caffeic acid hexoside	341.0878	341.087	C_15_H_18_O_9_	7.5	−2.36
7	3.17	Caffeoyltartaric acid	311.0409	311.041	C_13_H_12_O_9_	8.5	0.30
8	3.24	2-Isopropylmalic acid	175.0612	175.0612	C_7_H_12_O_5_	2.5	0.10
9	3.32	Catechin or Epicatechin	289.0718	289.0717	C_15_H_14_O_6_	9.5	−0.03
10	3.67	Coumaric acid hexoside isomer 1	325.0929	325.093	C_15_H_18_O_8_	7.5	0.49
11	3.69	Caffeic acid	179.035	179.0352	C_9_H_8_O_4_	6.5	1.50
12	3.71	Procyanidin B1 or B2	577.1351	577.1352	C_30_H_26_O_12_	18.5	0.07
13	3.79	Coumaroyltartaric acid isomer 1	295.0459	295.0461	C_13_H_12_O_8_	8.5	0.37
14	3.90	Ferulic acid pentoside	325.0929	325.093	C_15_H_18_O_8_	7.5	0.39
15	3.93	Catechin or Epicatechin	289.0718	289.0718	C_15_H_14_O_6_	9.5	0.27
16	4.38	Myricetin-3-*O*-galactoside	479.0831	479.0828	C_21_H_20_O_13_	12.5	−0.67
17	4.49	Syringic acid	197.0455	197.0456	C_9_H_10_O_5_	5.5	0.32
18	4.80	Isoquercitrin	463.0882	463.0881	C_21_H_20_O_12_	12.5	−0.20
19	4.85	Quercetin 3-*O*-glucuronide	477.0675	477.0675	C_21_H_18_O_13_	13.5	0.07
20	5.59	p-Hydroxybenzoic acid	137.0244	137.0245	C_7_H_6_O_3_	5.5	0.31
21	5.20	Isorhamnetin 3-glucoside	477.1038	477.1038	C_22_H_22_O_12_	12.5	−0.12
22	6.22	Quercetin	301.0354	301.0356	C_15_H_10_O_7_	11.5	0.57
23	6.94	Kaempferol	285.0405	285.0408	C_15_H_10_O_6_	11.5	1.08

**Table 2:** Table demonstrating the parameters tested through different examinations, their validation and scaling.

FOB Test	Tested Parameter	Validation	Scale
**Home-cage measurements**			
Body position	General condition, pain	R	1 to 3
Respiration	Respiration rate, weakness	R	1 to 6
Vocalization	Pain	Y/N	
Palpebral closure		R	1 to 3
**Hand-held observations**			
Reactivity		R	1 to 5
Handling		R	1 to 4
Palpebral closure		R	1 to 3
**Open field activity**			
Number of rearings	Exploratory activity	N	
Gait	Posture	R	1 to 6
Arousal	Activity over time	R	1 to 6
Defecations number	Autonomic system function	N	
Diarrhea	Autonomic system function	Y/N	
Urinations number	Autonomic system function	N	
Stereotypical behavior		Y/N	
**Sensorimotor reflexes**			
Approach response		R	1 to 4
Touch response		R	1 to 4
Eyelid reflex		Y/N	
Sound response		R	1 to 3
Tail pinch response	Pain response/locomotion	R	1 to 4
Righting reflex	Proprioception	R	1 to 4
Contact placing response	Corticospinal system function/Proprioception	R	0 to 2
Crossed extensor reflex	Corticospinal system function/Proprioception	R	1 to 2
**Sensorimotor measurements**			
Footprint/gait analysis			
Stride length	Ataxia, coordination, locomotion	M	
Stride width	Base of support, locomotion	M	
Foot rotation (R)	Sciatic nerve function	M	
Foot rotation (L)	Sciatic nerve function	M	
Interpedal distance	Ataxia, coordination, locomotion	M	
Landing foot splay	Vestibular and proprioceptive sensation/motor efferent fibres	M	
Grip strength	Touch and proprioceptive sensation/motor efferent fibres	M	
Sticky paper	Touch receptors/motor efferent fibres	M	
Von Frey hair pinch test	Mechanoreceptors, pain receptors/motor efferent fibres	M	

(R = ranking system, M = measured value, Y/N = Yes or No declaration).

**Table 3:** Mean body weight of each group at four studied time points.

Group	Pre-Treatment	Mid-Treatment	End-Treatment	Post-Treatment
**Control (group A)**	213 ± 15.62	228 ± 12.5	225 ± 9.75	232 ± 9.3
**Group B**	196 ± 10.77	201 ± 11.77	207 ± 11.79	221 ± 4.3
**Group C**	226 ± 10.77	220 ± 10.25	218 ± 10.07	227 ± 15.46
**Group D**	199 ± 1.05	202 ± 10.9	192.5 ± 7.77	212.5 ± 6

**Table 4:** Effect of oxaliplatin with and without GPE treatment on gait analysis measurements at the end of oxaliplatin administration.

	Group A	Group B	Group C	Group D
Foot rotation R	7.9 ± 1.63	11.15 ± 1.63	8.3 ± 1.4	11.68 ± 1.44
Foot rotation L	10.7 ± 1.9	11.85 ± 1.9	11.57 ± 1.6	12.51 ± 1.65
Stride length R	10.65 ± 0.44	11.08 ± 0.35	11.03 ± 0.3	10.9 ± 0.33
Stride length L	10.81 ± 0.42	11.04 ± 0.32	10.98 ± 0.34	11.33 ± 0.29
Stride width	4.49 ± 0.2	4.35 ± 0.21	4.15 ± 0.16	4.13 ± 0.17
Interpedal distance	5.47 ± 0.2	5.5 ± 0.19	5.5 ± 0.15	5.48 ± 0.16

**Table 5:** Values of the four sensorimotor tasks employed for all animal groups at the end of the oxaliplatin treatment period for the evaluation of the oxaliplatin treatment and the consecutive GPE administration (N.S. = not statistically significantly different).

Sensorimotor Tasks	Group A	Group B	Group C	Group D	
Landing foot splay (cm)	3.99 ± 0.5	4.35 ± 0.34	4.43 ± 0.29	4.58 ± 0.28	N.S.
Grip strength (N)	3.11 ± 0.18 *	2.81 ± 0.18 **/***	3.4 ± 0.15 **	3.53 ± 0.15 */***	* *p* ≤ 0.1 ** *p* ≤ 0.05 *** *p* ≤ 0.01
Sticky paper (sec)	157.7 ± 24	124.9 ± 24.3	126.8 ± 19	161.1 ± 18.5	N.S.
Von Frey hair pinch test (grams)	0.40 ± 0.0 *	1.04 ± 0.3 */**/***	0.43 ± 0.03	0.42 ± 0.02	* *p* ≤ 0.05 ** *p* ≤ 0.05 *** *p* ≤ 0.05

**Table 6:** TAC and TBARS levels in control group (group A) and animals treated with oxaliplatin only (group B) or oxaliplatin and GPE (group C, D).

	TAC (mmol DPPH/L Plasma)	SEM	TBARS (μmol/L Plasma)	SEM
group A	0.77	0.048	48.4	2.11
group B	0.77	0.006	40.3	1.07
group C	0.79	0.076	47.8	5.14
group D	0.67	0.012	40.4	4.68

**Table 7:** Morphometrical results of the DRG neurons.

Morphometric Parameters	Group A	Group B	Group C	Group D	*p*
Total DRG Neuron Number ^c^	14.138 ± 1.590.37	17.020 ± 2.278.97	15.090 ± 2.0000	16.782 ± 1.194.75	N.S.
Large DRG neurons ^b^	10.4%	2.8%	3.3%	3.8%	-
Medium DRG neurons ^b^	30.8%	24.1%	21.2%	26%	-
Small DRG neurons ^b^	58.8%	73.2%	75.6%	70.2%	-
Mean somatic Area (μm^2^) ^a^	526.96 ± 4.24 *	390.94 ± 2.48 *^/^**	393.92 ± 3.39	428.87 ± 2.82 **	*^,^** *p* < 0.01
Area of Large DRG neurons (μm^2^) ^a^	1.322.18 ± 10.54 */**	1.196.91 ± 12.48 *^/^***	1.275.81 ± 21.35 ***	1.237.48 ± 13.27 **	*^,^** *p* < 0.001 *** *p* < 0.01
Diameter ^a^	24.13 ± 0.48 *^/^**	20.43 ± 0.79 *	20.32 ± 0.65 **	21.49 ± 0.38	*^,^** *p* < 0.01
Perimeter ^a^	86.25 ± 1.61 *^/^**^/^***	71.97 ± 2.44 *	72.19 ± 3.21 **	75.26 ± 1.88 ***	*^,^** *p* < 0.01 *** *p* < 0.05

^a^ Data expressed as Mean Values ± S.E.M; ^b^ Data expressed as Percentages %; ^c^ Mean values after the use of c.f., N.S. = not statistically significant.

## Data Availability

Data is available through communication with the corresponding author.
